# High-Grade Cervical Lesions Among Women Attending A Reference Clinic In Brazil: Associated Factors And Comparison Among Screening Methods

**DOI:** 10.1371/journal.pone.0102169

**Published:** 2014-07-11

**Authors:** Neide T. Boldrini, Luciana B. Freitas, Amanda R. Coutinho, Flavia Z. Loureiro, Liliana C. Spano, Angélica E. Miranda

**Affiliations:** Post-Graduate Program in Infectious Diseases, Federal University of Espirito Santo (Universidade Federal do Espírito Santo – UFES), Vitória-Espírito Santo (ES), Brazil; Shanghai Jiao Tong University School of Medicine, China

## Abstract

**Background:**

Although screening for cervical cancer is recommended for women in most countries, the incidence of cervical cancer is greater in developing countries. Our goal was to determine the prevalence and factors associated with high-grade lesions/cervical cancer among women attending a reference clinic in Brazil and evaluate the correlation of histology with cytology, colposcopy and the high-risk HPV (HR-HPV) tests.

**Methods:**

A cross-sectional study of women attending a colposcopy clinic was carried out. The patients were interviewed to collect demographic, epidemiological and clinical data. Specimens were collected for cervical cytology, *Chlamydia trachomatis* and HPV testing using the Hybrid Capture (HC) and PCR tests. Colposcopy was performed for all patients and biopsy for histology when cell abnormalities or cervical intraepithelial neoplasia (CIN) were present.

**Results:**

A total of 291 women participated in the study. The median age was 38 years (DIQ: 30–48 years). The prevalence of histologically confirmed high-grade lesions/cervical cancer was 18.2% (95%, CI: 13.8%–22.6%), with 48 (16.5%) cases of CIN-2/CIN-3 and 5 (1.7%) cases of invasive carcinoma. In the final logistic regression model, for ages between 30 and 49 years old [OR = 4.4 (95%: 1.01–19.04), history of smoking [OR = 2.4 (95%, CI: 1.14–5.18)], practice of anal intercourse [OR = 2.4 (95%, CI: 1.10–5.03)] and having positive HC test for HR-HPV [OR = 11.23 (95%, CI: 4 0.79–26, 36)] remained independently associated with high-grade lesions/cervical cancer. A total of 64.7% of the cases CIN-3\Ca in situ were related to HPV-16. Non-oncogenic HPV were only found in CIN-1 biopsy results. Compared to histology, the sensitivity of cytology was 31.8%, the specificity 95.5%; the sensitivity of colposcopy for high-grade lesions/cervical cancer was 51.0%, specificity was 91.4% and the concordance with HPV testing was high.

**Conclusions:**

The results confirm an association of HR-HPV with precursor lesions for cervical cancer. These data emphasize that cytological screening to detect precursor lesions is still important in some regions and that HR-HPV should be included for screening.

## Introduction

Although screening for cervical cancer, with varying age ranges and periodicity of testing, is recommended for women in most countries, the incidence of cervical cancer and its related mortality is far greater in developing countries [1] [2]. The use of cytological screening to detect and remove precursor lesions has had a huge impact on both the incidence and mortality of cervical cancer [1] [3]. However, despite the general decline in the incidence of squamous cell carcinoma (SCC) in some countries where organized or opportunistic cytology screening has been implemented [1] [2] [4] [5], cervical cancer still occurs [6]. This may be due to methodological limitations of screening and/or screening coverage [3] [7] [8].

Persistent human papillomavirus (HPV) infection with specific high-risk (HR) HPV is an indicator of high-grade squamous intraepithelial lesions (HSIL) [9]. HPV genotyping assays may be used in cytology negative, and in HPV positive women over 30 years old in the same manner as HPV triage testing is currently employed in women with atypical squamous cells of unknown significance (ASC-US) or borderline and mild dysplasia (BMD) cytology [10]. The aim of type-specific detection of HPV is to further stratify women with normal cytology who are HPV positive into different risk categories [11]. The identification of infection with HR-HPV among these women justifies immediate colposcopy [11]; also women with other HR-HPV infections can be managed less aggressively at lower risk [12] and moreover type-specific detection provides physicians with actionable information to treat the highest risk patients immediately [10].

Invasive cervical cancer (ICC) is the second most common cancer in women of childbearing age in Brazil and about 14.1% of women in the general population are estimated to be infected by cervical HPV infection at any given time [6] [13]. An estimated 17,540 new cases of ICC were diagnosed in Brazil in 2012 [13].

The best method for cervical cancer screening is still an uncertain decision in developing countries. Recommendations include several possibilities from those available, such as cytology, colposcopy, histology and HPV tests or even serial cytology [14] [15]. In Brazil, screening is performed by the tripod method - cytology followed by colposcopy and biopsy for histology when abnormalities are diagnosed in cytology [13].

Vitoria is the capital of the state of Espírito Santo, which covers an area of approximately 6,750 square miles on the southeastern coast between Rio de Janeiro and Bahia in Brazil. The economy is based on steel production, ports and harbors, agriculture, small industry, and tourism. The population of Espírito Santo is approximately 3.2 million, with the majority living in the metropolitan area of the capital. Among the total of estimated cases of ICC in Brazil, 340 cases are in Espiírito Santo State and 40 in Vitoria (State Capital), resulting in an incidence of 17.0, 18.7 and 20.3/100,000 women, respectively [13].

The goal of this study was to determine the prevalence and associated factors of high-grade lesions/cervical cancer among women attending a colposcopy clinic in Vitoria, Brazil. This study also evaluated the different screening methods used for this diagnosis.

## Methods

### Ethical Aspects

This study was submitted to and approved by the internal review board of the *Universidade Federal do Espírito Santo* (#086/09). Written consent was given by the patients for their information to be stored in the hospital database and used for research. They received counseling and treatment for diagnosed cervical disorders.

### Study Design

This is a cross-sectional study of women attending a reference clinic for colposcopy in a University Hospital in Vitoria, Brazil. This clinic is one of the main clinics for colposcopy care in the region.

### Population

All women attending the clinic between March and December 2011, presenting incident cytological lesions, were invited to participate in the study. A 30-minutes face-to-face interview was conducted with the use of a standardized questionnaire. The trained staff from the local health department conducted the interviews. Enrolled patients answered the face-to-face interviews which included demographic (age, schooling, marital status, family income); behavioral (tobacco use, alcohol and illicit drug use, age at first sexual intercourse, number of sex partners (lifetime and in the past year), types of sexual activity, frequency of condom use in the last year and STI history) and clinical data (previous pregnancy, age at first pregnancy, number of children).

They also underwent a gynecological evaluation and cervical scrape samples were collected for cytological analysis and the hybrid capture (HC) test for HPV and *Chlamydia trachomatis* (CT). A colposcopy examination was performed on all patients, independently if they presented cervical intraepithelial neoplasia (CIN) or not. Histological analyses were performed in face of any cytological or colposcopy abnormalities as defined by the Gynecology and Obstetrics Brazilian Society. Only one certified professional performed the colposcopy exams using Olympus equipment, model OCS-3. Women, who were pregnant, presented recurrent lesions, were HIV-infected or had been admitted with a previous diagnosis of invasive cervical cancer were excluded from the study.

### Laboratory Procedures

The HC tests were used for the detection of high-risk and low-risk genital HPV-DNA types and *Chlamydia trachomatis*. The HC test for HPV includes probes for the following HR-HPV: 16, 18, 31, 33, 35, 39, 45, 51, 52, 56, 58, 59, 68 and low-risk HPV (LR-HPV): 6, 11, 42, 43 and 44. HPV-DNA also was investigated by polymerase chain reaction (PCR), using the protocol previously established, which detects a fragment of 450 bp of the L1 gene region with consensus primers MY09/11 and was followed by the Restriction Fragment Length Polymorphism (RFLP).

### Sample Size Calculation

Sample size for this study was calculated to estimate the prevalence of high-grade lesions or cervical cancer diagnosed by the tripod diagnosis that is cytology plus colposcopy plus histology. The tripod method was considered the outcome (dependent variable). A 5% prevalence rate with confidence limits ±2.5% was taken as the basis for calculating the sample size [16]. A total of 280 women were needed, and thus it was planned to recruit a total of 322 to meet this requirement under the assumption of a 15% refusal rate.

### Statistical Analyses

Data were analyzed using the SPSS - statistical software program for Windows, version 17.0. A preliminary analysis was performed using exploratory techniques on the data. Chi-square tests and Student’s-t tests and variance analysis were used. The odds ratio (OR) was used as a measure of association, estimated with a 95%CI. For assessing ordinary variables we used the *chi-squared* test for trend to compare cases diagnosed by the tripod diagnosis (dependent variable) and the other methods (independent variables), negative result, LSIL and HSIL. Multivariate analysis was performed to estimate effects of independent variables, through the use of logistic regression models. Variables with p value≤0.15 entered in the multivariate logistic regression model. Variables were considered as significant when p value was ≤0.05. In order to determine how useful the test is to detect cervical lesions in this population, we calculate the sensitivity and the specificity of tests. Histology was used as the gold standard.

## Results

A total of 291 women (90.7%) out of the 322 recruited were included in the study. Patients excluded were: 7 (clinical invasive cancer); 5 (pregnancy); 10 (recurrent infections), 4 (HIV-infected), 4 (other immunosuppression conditions). The excluded patients presented similar characteristics than patients included in the study.

The median age of women was 38 years (Interquartile range: 30–48). The prevalence of high-grade lesions/cervical cancer was 18.2% (CI95%: 13.8%–22.6%), 48 (16.5%) cases of high-grade lesions (CIN-2, CIN-3\carcinoma in situ) and 5 (1.7%) subclinical cases of invasive cancer. The cancer cases came into the study as LSIL or HSIL and were diagnosed after histology in the colposcopy clinic.

A total of 74 (25.4%) women had up to four years of schooling, 178 (61.2%) were married and 83 (28.5%) had monthly incomes of three Brazilian minimum salaries (US $822.00) or less. Table 1 shows the demographic data; there was difference between groups only regarding age (p = 0.037).

**Table 1:** Demographic and behavioral characteristics of women attending a colposcopy clinic in Vitoria, Brazil, 2011 (N = 291).

	High-grade lesion^1^/cervical cancer		
Variables	Positive n (%) 53 (18.2)	Negative n (%) 238 (81.8)	p-value
**Age in years**			0.037
19–29	11 (20.8)	60 (25.2)	
30–49	36 (67.9)	119 (50.0)	
> = 50	6 (11.3)	59 (24.8)	
**Schooling in years**			0.684
0–4	16 (30.2)	58 (24.4)	
5–8	13 (24.5)	61 (25.6)	
> = 9	24 (45.3)	119 (50.0)	
**Marital status**			0.065
Single	10 (18.9)	54 (22.7)	
Married/living together	39 (73.6)	138 (58.0)	
Divorced/widow	4 (7.5)	46 (19.3)	
**Monthly income**			0.161
Up to 3 BMI*	42 (79.2)	166 (69.7)	
More than 3 BMI	11 (20.8)	72 (30.3)	
**Smokers**			0.042
Yes	26 (49.1)	81 (34.0)	
No	27 (50.9)	157 (66.0)	
**Illicit drug abuse**			0.121
Yes	4 (7.6)	7 (2.9)	
No	49 (92.4)	231 (97.1)	
**Age at first intercourse**			0.070
<15 years	11 (20.8)	27 (11.3)	
> = 15 years	42 (79.2)	211 (88.7)	
**# of partners/life**			0.089
More than one	45 (84.9)	176 (73.9)	
Only one	8 (15.1)	62 (26.1)	
**# of partners/last year**			0.140
More than one	1 (1.9)	19 (8.0)	
Only one	52 (98.1)	219 (92.0)	
**Condom use**			0.878
No	41 (77.4)	182 (76.5)	
Yes	12 (22.6)	56 (23.5)	
**Anal sex practice**			0.003
Yes	26 (49.1)	63 (26.5)	
No	27 (50.9)	175 (73.5)	
**Previous STI**			0.317
Yes	6 (11.3)	40 (16.8)	
No	47 (88.7)	198 (83.2)	

*BMI = Brazilian Monthly Income in 2011 = US $274.

1 Histologically confirmed ≥ CIN-2.

Behaviors are also described in Table 1: 108 (37.1%) were smokers 11 (3.8%) reported use of illicit drugs, 38 (13.1%) had first sexual intercourse before they were 15 years old, 221 (75.9%) had more than one partner in life, 220 (75.6%) reported not using condoms, 90 (30.2%) reported anal sex and 46 (15.8%) previous sexually transmitted infections (STI). Smokers (p = 0.042) and anal sex practice (p = 0.003) were more frequent among women with high-grade lesions/cervical cancer.

Clinical data are described in Table 2. A total of 286 women were tested by HC (Hybrid capture) for CT and HPV; HPV was also tested by PCR. Samples were not viable in five cases but they were included in the study because they had data from the tripod method. HC for *Chlamydia trachomatis* was positive in 4.9% (14/286). Having 4 or more children (p = 0.030), positive PCR for HPV (p = 0.001) and HR-HPV in HC (p = 0.001) were factors associated to high-grade lesions/cervical cancer.

**Table 2:** Clinical characteristics of women attending a colposcopy clinic in Vitoria, Brazil, 2011 (N = 291).

	High-grade lesion/cervical cancer		
Variables	Positive n (%) 53 (18.2)	Negative n (%) 238 (81.8)	p-value
**Previous pregnancy**			0.094
Yes	49 (92.5)	195 (81.9)	
No	4 (7.5)	43 (18.1)	
**Age at first pregnancy** *			0.997
<15 years	1 (2.0)	4 (19.7)	
> = 15 years	48 (98.0)	191 (80.3)	
**# of children**			0.030
None	5 (9.4)	48 (20.2)	
1–3 children	33 (62.3)	154 (64.7)	
> = 4 children	15 (28.3)	36 (15.1)	
**Chlamydia test** **			0.478
Positive	2 (3.8)	12 (5.2)	
Negative	51 (96.2)	221 (94.8)	
**PCR for HPV** **			0.001
Positive	47 (88.7)	112 (48.1)	
Negative	6 (11.3)	121 (51.9)	
**High risk HPV (HC)** **			0.001
Positive	44 (83.0)	76 (32.6)	
Negative	9 (17.0)	157 (67.4)	
**Low risk HPV (HC)** **			0.435
Positive	6 (11.3)	19 (8.2)	
Negative	47 (88.7)	214 (91.8)	

*Referent to 244 women who report pregnancy;

**Referent to 286 women tested by HC (Hybrid capture) for *Chlamydia trachomatis* and HPV and patients tested by PCR for HPV.

In the multivariate analyses, being 30–49 years old [OR = 4.4 (CI95%: 1.01–19.04); a tobacco user [OR = 2.4 (CI95%: 1.14–5.18)]; having anal sex [OR = 2.4 (CI95%: 1.10–5.03)] and being HR-HPV in HC [OR = 11.2 (CI95%: 4.79–26.36)] were factors associated to high-grade lesions/cervical cancer.

Table 3 describes the association between screening methods. Isolated cytology and cytology plus colposcopy presented a high rate of concordance with the tripod diagnosis for negative results (94.4%) and the inclusion of the HR-HPV test increase these concordance to 96.4%. Regarding positive cases, the inclusion of the HR-HPV improved the diagnoses of HSIL/Ca in situ and ICC, diagnosing all cases except one. These results were not statistically significant.

**Table 3:** Association between tripod diagnostic and cytology, colposcopy and HR-HPV test among women attending a colposcopy clinic in Vitoria, Brazil, 2011 (N = 291).

		Cervical lesions using tripod diagnosisfor diagnosis* N (%)				*P value*
	Negative196 (67.4)	CIN-142 (14.4)	CIN-230 (10.3)	CIN-3/Ca in situ18 (6.2)	Invasive Carcinoma5 (1.7)	
**Cytology**						0.590
Negative	68 (94.4)	3 (4.2)	1 (1.4)	0 (0)	0 (0)	
LSIL	124 (73.4)	23 (13.6)	14 (8.3)	7 (4.1)	1 (0.6)	
HSIL	4 (8.0)	16 (32.0)	15 (30.0)	11 (22.0)	4 (8.0)	
**Cytology + colposcopy**						0.328
Negative	67 (94.4)	3 (4.2)	1 (1.4)	0 (0)	0 (0)	
LSIL	122 (76.3)	22 (13.8)	12 (7.5)	4 (2.5)	0 (0)	
HSIL	7 (11.7)	17 (28.3)	17 (28.3)	14 (23.3)	5 (8.3)	
**Cytology + colposcopy + HR − HPV**						0.079
Negative	53 (96.4)	1 (1.8)	1 (1.8)	0 (0)	0 (0)	
LSIL	91 (86.7)	11 (10.5)	2 (1.9)	1 (1.0)	0 (0)	
HSIL	52 (39.7)	30 (22.9)	27 (20.6)	17 (13.0)	5 (3.8)	

*Gold standard method (Tripod diagnostic: Cytology + Colposcopy + Histology).

There are some data not shown on Tables. On comparing isolated tests (cytology, colposcopy and HR-HPV test) to cases with histology, the sensitivity of cytology was found to be 31.8% and the specificity 95.5%. When analyzing the findings of colposcopy compared with histology results, abnormal colposcopy findings were found in 90.4% of the cases of histology abnormalities and 45% of the findings of the biopsies of women with normal histology. The sensitivity of colposcopy for high-grade lesions/cervical cancer compared to histology was 51.0% and specificity was 91.4%. Regarding the positivity of HPV, 55.9% of patients had a positive HPV test.

Among the 154 cases of DNA-HPV identified by PCR, 120 (75%) were infected by high-risk genotypes. HPV-16 was identified in 35 (22.7%) from all HPV cases, followed by HPV-31 in 7 (4.5%), HPV 35, 52 and 58 in 6 (3.9%), HPV-18 and 62 in 4 (2.6%), HPV-6, 26, 33, 53, 82 and 89 in 3 (1.9%), HPV-51, 66, 67, 73 and 84 in 2 (1.3%) and finally HPV-11, 42, 45, 56, 59, 61, 69, 70, 72 and 92 in 1 (0.6%). Of the 160 genotypes detectable by HC, 120 (75%) are HR-HPV and 40 (25%) of LR-HPV.

A total of 64.7% of the cases of CIN-3\Ca in situ were related to HPV-16. The non-oncogenic HPV-6 and 11 were only found in CIN-1 biopsy results. Five cases were of invasive carcinoma: two were positive for HPV-16, one for HPV-18, one for HPV-31 and one for HPV-73.

## Discussion

This study reports a prevalence rate of high-grade lesions/cervical cancer of 18.2% among women attending a reference clinic for colposcopy in Vitoria, Brazil. This rate is higher than that reported in a Brazilian systematic review that included three large studies showing a prevalence of high-grade intraepithelial lesion of 6–12% [17]. However, the present study is in agreement with a study performed in a colposcopy clinic in Rio de Janeiro (19.3%) [18]. Both these clinics receive patients that have been referred for abnormal cytology and colposcopy as well as for treatment of precursor lesions of cervical cancer.

Factors associated with high-grade lesions in this study were age between 30–49 years old, tobacco users, anal sex practice and HR-HPV. HPV infection is described as a necessary cause for ICC [19]. Although HPV-16 was the most frequent type in this study, its frequency was lower than that reported in the scientific literature. Additional risk factors, in part related to the host, are involved in the progression of HPV infections to carcinoma in situ and cancer and they include smoking, hormonal contraceptive use, multiple pregnancies and coinfection with other STI agents such as herpes simplex virus-2 (HSV) and *Chlamydia trachomatis* [20] [21] [22]. Factors related to the virus, such as the HPV type involved in the infection, viral variants, persistence of infection and viral load, also contribute to the progression of the infection to cancer, [20] [23]. The HR-HPV types are more likely to cause persistent lesions and be associated with precancerous lesions. It is estimated that high-risk HPV 16 and 18 causes around 70% of all cervical cancers worldwide [24] [25], with prevalence rate ranges between 50 and 70% for HPV-16 [22] [24].

There is evidence that HPV is the principal etiological agent in cervical neoplasia, and some other sexually transmitted agents may either contribute to cervical carcinogenesis, such as herpes simplex virus (HSV), CT and human immunodeficiency virus (HIV). Epidemiological studies suggested that HSV may have a role in cervical neoplasia, but there is no clear supportive experimental evidence. However, longitudinal epidemiological studies have provided evidence that CT is an independent risk factor for the development of cervical squamous carcinoma and this association is serotype specific. The increased risk of cervical neoplasia in patients infected with HIV also has been recognized for over a decade and HIV may interact with HPV either by alternating the HPV gene transcription or by immunosuppression [21]. CT was not associated to HSIL or cervical cancer in the present study, HSV was not included because of methodological issues and HIV infection was an exclusion criteria.

In the present study, positive HPV tests were found in 76.9% of the patients with CIN-1, 82.75% with CIN-2, 94.44% with CIN-3 or Carcinoma in situ and 100% of patients with invasive carcinoma. Almost 70% of the cases with CIN-3\carcinoma in situ were related to HPV-16. HPV types 6 and 11 were only found in cases of CIN-1. A study performed in Rio de Janeiro reported a 50.1% prevalence of HPV, ranging from 25% among women with normal cytology to 100% in women with high-grade intraepithelial lesions [26]. A similar study in Venezuela showed that HPV-DNA was present in 68% of patients with low SIL, 95% with high SIL and 98.7% with cervical cancer [27].

The distribution of HPV types differs between cervical adenocarcinoma and SCC of the cervix, HPV-16 accounts for the majority of SCC, both HPV-16 and HPV18 are prominent in adenocarcinoma [23] [28]. However, few such cases (1.7%) were detected in the present study. Another Brazilian study has also reported relatively low frequency of HPV-18 in asymptomatic young women attending public schools in Rio de Janeiro [25].

The association of cytology, colposcopy, and histology is called the “tripod diagnosis” and allows the diagnosis of neoplastic and pre-neoplastic lesions in 90% of cases [29]. This is important, because in the literature, estimates of sensitivity and specificity of cervical cytology in women generally have different results [3] [30] [31] [32]. For women in general, some meta-analysis studies estimated that the sensitivity of cytological approaches 55% [3] [33] 34]. As in this study, which found a sensitivity of 31.8% and specificity of 95.5%, Fox and colleagues also found low sensitivity and high specificity of cytology studying HIV-infected women in Rio de Janeiro [32].

Although a cross-sectional study is not the best study for determining risk factors, its application is justified for assessing the prevalence of and the associated factors for HR-HPV among women attending the colposcopy clinic. In this study, we cannot rule out of the possibility of response bias because there is always a general tendency to give socially acceptable answers regarding risk factors. Nevertheless, it is important to emphasize the importance of performing cervical cytological exams to monitor women’s health routinely and any degree of abnormality in this test should be referred for colposcopy assessment [35] [36].

Cytology, colposcopy and histology are three important and complementary methods to study the cervix and its injuries. Different findings can show discrepancies among results, which can be explained by the experience of the pathologists and the resolution of the colposcopy. The strategies for diagnosing cervical lesions it is important theme to be discussed in Brazil because the cytology is recommended for screening. If our results are not satisfactory we need to start including other methodologies. The inclusion of HR-HPV test as a complementary test can assist in the diagnosis of cervical lesions identifying high-risk cases.

Cancer prevention programs exist and have been shown to be successful at avoiding disease progression. Although this is encouraging, much work remains to identify additional innovative interventions that address the social, cultural, and environmental influences of HPV infection and cervical cancer. There is also a need to find better ways of disseminating evidence-based approaches to cervical cancer prevention, so that effective interventions are more widely used. Sexually active women need access to confidential, low-cost, and friendly services to teach them how to protect themselves.
